# Genetic mapping of pollen fertility restoration QTLs in rye (*Secale cereale* L.) with CMS Pampa

**DOI:** 10.1007/s13353-020-00599-9

**Published:** 2021-01-07

**Authors:** Agnieszka Niedziela, Waldemar Brukwiński, Piotr Tomasz Bednarek

**Affiliations:** 1 Plant Breeding and Acclimatization Institute, NRI, Radzików, 05-870 Błonie, Poland; 2 DANKO Plant Breeding Ltd., Choryń 27, 64-000 Kościan, Poland

**Keywords:** Cytoplasmic male sterility, Rye, QTL mapping

## Abstract

Cytoplasmic male sterility (CMS) is a widely applied plant breeding tool for hybrid seed production. The phenomenon is often caused by chimeric genes with altered open reading frames (ORFs) located in the mitochondrial genomes and expressed as novel genotoxic products that induce pollen abortion. The fertility of CMS plants can be restored by nuclear-encoded genes that inhibit the action of ORFs responsible for pollen sterility. A recombinant inbred line (RIL) mapping population S64/04/01, encompassing 175 individuals, was used for genetic map construction and identification of quantitative trait loci (QTLs) responsible for fertility restoration in rye (*Secale cereale* L.) with CMS Pampa. The genetic map of all seven rye chromosomes included 15,516 SNP and silicoDArT markers and covered 1070.5 cm. Individual QTLs explaining 60% and 5.5% of the fertility trait’s phenotypic variance were mapped to chromosomes 4R (*QRft-4R*) and 5R (*QRft-5R*), respectively. Association mapping identified markers with the highest *R*^*2*^ value of 0.58 (*p* value = 2.21E-28). Markers showing the highest associations with the trait were also mapped to the 4R chromosome within the *QRft-4R* region. Based on marker sequence homology, putative genes involved in pollen fertility restoration were suggested. Five silicoDArTs were converted into PCR-based markers for further breeding purposes.

**Supplementary Information:**

The online version contains supplementary material available at 10.1007/s13353-020-00599-9.

## Introduction

Cultivated rye (*Secale cereale* L.) is a cross-pollinated, diploid (2n = 14) cereal with seven pairs of chromosomes. Due to its exceptional tolerance to low temperatures in winter and minimal soil requirements, rye can be cultivated in regions with severe climates or in those with light, sandy, low pH, and infertile soils (Bushuk 2001).

Rye breeding efforts are focused on improving grain yield as the most crucial objective. At present, hybrid rye varieties give 10–20% higher yields than open-pollinated varieties grown under the same agrotechnical conditions. One of the most exploited hybrid breeding systems in rye relies on Pampa (P) sterilizing cytoplasm, which originated from Iranian primitive rye and Argentinian landraces (Geiger and Schnell 1970; Geiger and Miedaner 1996). Other types of CMS, exploiting *Vavilovii* cytoplasm and designated as C, G, and R, are also studied extensively (Melz and Adolf 1991; Kobyljanskij, 1969; Börner et al. 1998; Łapiński and Stojałowski 2003; Stojałowski 2005; Milczarski et al. 2016). However, except for the G cytoplasm (Melz et al. 2001), these resources are not employed in commercial breeding programs.

Restoration of pollen production in sterile plants, including rye hybrids, requires a male parent (pollinator) that holds effective pollen fertility restoration (*Rf*) nuclear genes. Unfortunately, male fertility restoration genes for the Pampa cytoplasm appear in less than 5% of European rye materials, with effectiveness in the 2–74% range (Miedaner et al. 2005). Moreover, the activities of these genes may be dependent on the environmental conditions present before and during flowering (Geiger and Miedaner 1996). Lack of pollen and the resulting presence of young, unfertilized ovaries facilitate infection by the ergot fungus *Claviceps purpurea*, which replaces the seeds with dark mycelial masses sclerotia (Miedaner and Geiger 2015). Successful breeding of rye hybrids is determined by the identification of the major nuclear restorer QTLs. So far, mapping analyses have mapped the QTLs with highest restoration ability for CMS Pampa (*Rf*) to chromosomes 1RS in European rye resources (line L18) and 4RL in non-adapted rye accessions from Iran (IRAN IX) (*Rfp1*) and Argentina (Pico Gentario) (*Rfp2*) (Miedaner et al. 2000). The relevant gene on 1RS explained 54% of the phenotypic variation, and the gene on 4RL explained 68% and 59% of phenotypic variation in the IRAN IX and Pico Gentario populations, respectively. Another source of superior pollen fertility restoration is the *Rfp3* QTL derived from the Iranian primitive rye “Altevogt 14160,” which mapped within a 2.5 cm segment on the 4RL chromosome (Hackauf et al. 2017). Three minor QTLs from European line L18 were mapped to chromosomes 3RL, 4RL, and 5R and explained 17%, 9%, and 11% of the phenotypic variation, respectively (Miedaner et al. 2000). Additionally, in the Pico Gentario population, a gene that significantly enhanced the expression of the major restorer gene *Rfp2* was found on the 6R chromosome (Miedaner et al. 2000).

The *Rfp1* and *Rfp2* QTLs have been extensively transferred to pollinator elite inbred lines using the backcross breeding method, raising pollen fertility to 55–90% (Miedaner et al. 2005). The marker-assisted selection (MAS) breeding process was streamlined by the development of sequence-characterized amplified region (SCAR) markers based on RAPD and AFLP markers tightly linked to the critical QTLs (Stracke et al. 2003). However, long intervals between the markers flanking the *Rfp1* (2.9 cm) and *Rfp2* (5.2 cm) QTLs are problematic because the region may encode undesirable genes surrounding the introgressed allele (Frisch and Melchinger, 2000; Miedaner et al. 2017). Recent development of accurate conserved ortholog set (COS) markers (TC300731 and TC256739) delimiting *Rfp1* within a 0.7 cm interval (Hackauf et al. 2012) and an EST-derived CAPS marker, c28385, which co-segregates with *Rfp3* (Hackauf et al. 2017), was also beneficial. These markers allowed analysis of linkage drag effects between the restorer gene and undesirable gene(s)/QTLs and facilitated investigation of orthologs of the *Rf* QTLs originating from different genetic rye resources, as well as from barley (Hackauf et al. 2017).

High-density genetic maps are vital to increase the precision of QTL mapping and marker development for efficient MAS programs. Several maps have been constructed for rye. One early map uses low-throughput RFLPs (Korzun et al. 1998; Ma et al. 2001). Another map relies on AFLP and RAPD markers (Masojć et al. 2001; Saal and Wricke, 2002; Bednarek et al. 2003; Milczarski et al. 2007). The average saturation of the current rye maps varies, with a single marker per 3.0–4.0 cm (Korzun et al. 1998; Masojć et al. 2001; Ma et al. 2001; Bednarek et al. 2003). All of the maps have gaps that extend over more than 20 cm (Korzun et al. 1998; Ma et al. 2001; Saal and Wricke 2002; Bednarek et al. 2003). The introduction of SSR markers allowed the development of more saturated maps (Saal and Wricke 1999; Khlestkina et al. 2004; Hackauf and Wehling 2003; Milczarski et al. 2007). However, the introduction of DArT and later DArTseq technologies improved map saturation and reduced gap size to approximately 1.1 cm (Milczarski et al. 2016). There length of the maps were 1245 cm (Bauer et al. 2017), 1593.0 cm (Milczarski et al. 2011), and 3144.6 cm (Bolibok-Brągoszewska et al. 2009). The DArT-based genetic map enriched with GBS markers was also devoted to the localization of the *Rfc1* gene that restores male fertility in rye with the C source of sterility-inducing cytoplasm (Milczarski et al. 2016). Recently, Bauer et al. (2017) used a whole-genome shotgun (WGS) sequencing strategy to create a high-density genetic map of rye inbred line Lo7 with an average distance of 0.6 cm between loci.

Despite numerous studies devoted to the pollen fertility restoration trait in rye (Miedaner et al. 2000, Stracke et al. 2003, Hackauf et al. 2012, Miedaner et al. 2017), little is known regarding the genes participating in the phenomenon and their roles in the trait. Based on data from other species, *Rf* genes may belong to the pentatricopeptide repeat (PPR)-containing gene family (Wang et al. 2006, Klein et al. 2006, Kazama and Toriyama 2003, Brown et al. 2003, Desloire et al. 2003) that encodes proteins required for many posttranscriptional processes in organelles (reviewed in Hammani and Giegé, 2014). In most cases, *Rf*-PPR proteins prevent the accumulation of the CMS-gene products (Kazama et al. 2008, Uyttewaal et al. 2008, Hu et al. 2012). The other candidate gene implicated in fertility restoration belongs to the mitochondrial transcription termination factor family (mTERF) and was identified in rye (Hackauf et al. 2017) and barley (Bernhard et al. 2019). Fine-mapping of the 4R chromosome region carrying *Rfp1*, *Rfp2*, and *Rfp3* loci demonstrated its orthology to sub-genomic regions in rice and *Brachypodium* that contained mTERF and PPR-encoding genes (Hackauf et al. 2012 and 2017).

The study aimed to identify QTLs responsible for male fertility restoration in rye with CMS Pampa using an advanced recombinant inbred line (RIL) population utilizing high-throughput DArTseq marker technology for genetic map construction and evaluation of sequence-specific markers linked to pollen fertility restoration trait. Furthermore, we were also interested in identifying plausible genes participating in pollen fertility restoration in rye with CMS Pampa.

## Materials and methods

### Plant materials

The RIL F7 mapping population S64/04/01, encompassing 175 individuals, was developed in DANKO Plant Breeding Ltd. (Breeding Department Choryń, Poland) by a single seed descent method from a biparental cross with female parent S305N/00 (maintainer) on non-sterilizing cytoplasm and male parent SO37R/05 on CMS Pampa (restorer line). The F2 seeds obtained from bag-isolated F1 plants were ground-planted and used to develop the RIL mapping population up to the F7 generation.

### Phenotyping

Pollen fertility restoration of the RILs was evaluated indirectly via phenotyping of BC1F1 materials derived via backcrossing of maternal line S305P/00 (on CMS Pampa) and RIL F7 lines of mapping population S64/04/01. Individual BC1F1 progeny plants were grown in 2 m long single rows with 25 cm spacing during the 2016/17 vegetation season in a field belonging to the DANKO Plant Breeding Ltd., Breeding Department Choryń, Poland. The evaluation of male fertility was conducted in five plants via visual scoring of three spikes per plant at the flowering stage, according to the 1–9 bonitation scale developed by Geiger and Morgenstern (1975). Fully male sterile plants were scored as 1–3 and referred to non-dehiscent, empty anthers with decreasing levels of degeneration. Partly sterile plants exhibited values 4, 5, and 6 and differed in their percentages of male fertile anthers (< 10%, 11–50%, and < 50%, respectively). Plants with fully pollen-shedding anthers of increasing anther size were scored as 7–9.

The normal distribution of the fertility trait was tested using the Kolmogorov-Smirnov test implemented in XlStat software (XlStat 2019). The *χ*^*2*^ goodness-of-fit test was conducted to verify a trait segregation ratio of 1:1 using MapQTL 5 (Van Ooijen, 2004). In this calculation, the partially fertile plants were included in the fertile class.

### DNA isolation

Total genomic DNA was extracted from approximately 100 mg fresh leaf tissue from each line of the 175 RIL: S64/04/01 population using a DNeasy Plant Mini Kit 250 according to the manufacturer’s instructions. DNA integrity and purity were assessed via electrophoresis on 1% agarose gels stained with EtBr (0.5 μg/ml) in TBE buffer. DNA was quantified spectrophotometrically using a NanoDrop (ND-1000) instrument.

### Genotyping

The DArTseq platform developed by Diversity Arrays Technology Pty Ltd. (Canberra, Australia) was employed for genotyping. The platform detects SNP (single nucleotide polymorphisms) and silicoDArT markers using *Pst*I and *Taq*I digestions for the reduction of genome complexity followed by next-generation sequencing (NGS) of short fragments with a HiSeq 2000 sequencing system (Illumina Inc., San Diego, USA) (Sánchez-Sevilla et al. 2015). The resulting marker sequences were filtered for quality, with a cutoff value at 90% confidence. The SNP and silicoDArT markers were coded as “0” or “1,” according to their absence or presence, respectively.

### Linkage map construction

The genetic map was constructed using MultiPoint Ultra-Dense software (Ronin et al. 2015). Markers exhibiting > 15% missing data were excluded. All SNP and silico DArT loci that showed no or minimal deviation from the expected 1:1 segregation ratio (*χ*^*2*^ ≤ 19.2) were employed in the analysis.

Genetic map construction consisted of the following steps: (1) Markers with zero distance were grouped, and a “delegate” was selected from each group. Only markers with at least the same number of twins as the predefined threshold were selected as delegates and were defined as “skeleton.” Markers exhibiting identical segregation patterns as the delegate/skeleton markers were assumed redundant. (2) All remaining markers, except for candidate twins, were removed to the heap. (3) Delegate markers (most representative skeletons and their redundant markers) were clustered, and the resultant linkage groups (LGs) were ordered. (4) Gaps were filled, and LG ends were extended using markers from the heap (heap contains markers that due to, i.e., segregation problems or missings were primarily removed from mapping procedure). (5) Markers violating map stability and monotonic growth of distance from a marker and its subsequent neighbors were removed.

### Assigning linkage groups to the rye chromosomes

The LG groups were assigned to rye chromosomes based on known chromosomal locations of SNP and silicoDArT markers provided by Diversity Arrays Technology Pty Ltd. The S-L orientation of the LGs on rye chromosomes and the alignment of the LGs to the rye genome were verified using a high-density genetic map of rye inbred line Lo7 presented by Bauer et al. (2017). Similarities between SNP and silicoDArT marker sequences and the sequences of WGS contigs placed on the map were identified for this purpose. The order of common (homologous) markers was tested by Pearson correlation in XlStat software (XlStat, 2019). The map was visualized in MapChart (Voorrips, 2002).

### Quantitative trait loci (QTL) analysis

Relationships between the segregation of molecular markers and studied traits were analyzed using a nonparametric Kruskal-Wallis K* test (Lehmann, 1975) using the MapQTL package, version 5.0. (VanOoijen 2004). Genomic regions were considered to contain QTLs if the significance of molecular markers was *p* ≤ 0.005. Verification of QTL mapping was performed using the composite interval mapping (CIM) method with Windows QTLCartographer software, version 2.5 (Wang et al. 2007). QTL significance was evaluated using a 1000-permutation test with α = 0.05 significance level. The *R*^2^ value (phenotypic variance explained (PVE) by a QTL) was calculated as the proportion of each QTL’s phenotypic variation. A backward regression method with a window size of 3 cm, walk speed of 1 cm, and number of control markers equal to five was used for CIM.

### Population structure and association mapping

Population structure was investigated using principal components analysis (PCA) in PAST software (Hammer et al. 2001). Association mapping was performed in TASSEL (Bradbury et al. 2007) using all SNP and silicoDArT markers. The General Linear Model (GLM) was tested to evaluate markers associated with pollen fertility restoration genes. Significant associations were indicated by the Bonferroni test with *p* < 0.01 (0.01/number of markers). The degree of association was represented by the determination coefficient (*R*^*2*^).

### Marker sequence homology

The DNA sequences of 435 SNPs and silicoDArTs linked and/or associated with the trait were searched using BLASTn against GenBank in the National Center for Biotechnology Information (NCBI) database. Skeleton, redundant, and added (approximated/regressed on map) markers linked to the QTLs of the trait, and mapped on the 4R and 5R chromosomes, were searched. A similar analysis was performed for markers associated with the trait and their redundant counterparts based on segregation. Classification of the query sequences was based on (1) identity (I, percentage of similarity between the subject and query sequences over the length of the coverage area), (2) query cover (QC, percentage of the query sequence that overlaps the subject sequence), and (3) *E* value (probability value) criteria. The taxonomic category selected during searches was the *Poaceae* family.

Sequence similarity between markers and rye contigs (Bauer et al. 2017) was searched using the BLASTn program and Lo7 WGS contigs v2 database (https://webblast.ipk-gatersleben.de/ryeselect/).

### Conversion of SNP and silicoDArT markers to PCR-based assays

Twenty-seven SNP and silicoDArT markers linked to or/and associated with fertility restoration were converted into PCR-based assays (Suppl. File S1). DNA sequences of the markers were blasted against the sequences of rye Lo7 WGS contigs (Bauer et al. 2017, https://webblast.ipk-gatersleben.de/ryeselect/). The 69 bp marker sequences were contained within contigs (ca. 1000–7000 bp in size), which made it possible to design primers. For two markers (3,358,169 and 5,037,479), the primers were designed based directly on the sequence of the gene encoding keratin-associated protein (KAP) 5–4-like.

SNPs and silicoDArTs with a 99–100% match to the sequence of rye Lo7 WGS contigs (sequence identities = 68/69 or 69/69) were analyzed in Primer3web software version 4.1.0 (http://bioinfo.ut.ee/primer3/) to identify primer pairs for their amplification. The primer design criteria were as follows: 40–60% GC rich; minimum annealing temperature, 50 °C; no or negligible secondary structures; and product size ≥ 400 bp.

Converted markers were tested using DNA from fertile and sterile parents (SO37R/05 and S305P/00). The optimal annealing temperature was inferred using a gradient PCR with temperatures set between 51.0 and 65.0 °C (Labcycler Gradient, SensoQuest GmbH). Reaction mixtures consisted of 10 ng of total genomic DNA, 50 μmols each of PCR primer, 2.5 mM dNTPs, 2.5 mM MgCl_2_, 1 × reaction buffer, and 0.25 U of Gold HotStart DNA Polymerase (Syngen Biotech Ltd.) in a final volume of 10 μl. Amplification was performed using the following profile: [95 °C-15′] [95 °C-30″; X°C-45″; 72 °C-45″] × 35 [72 °C-10′] [5 °C ∞], where “X” reflects the annealing temperature selected from PCR gradient profile reactions (Suppl. File S1). The PCR products were separated on 1.2% agarose gels in TBE buffer at 5 V/cm for 1 h. Segregation of the PCR-based markers was tested on DNA samples of 48 lines (23 fertile; 3 partly fertile, and 22 sterile) from the S64/04/01 mapping population. PCR-based markers with segregation consistent with their DArTseq or silicoDArT counterparts were considered suitable for selection purposes. Markers that were successfully converted to sequence-specific PCR-based assays were appended with “c” at the end of their original counterpart names (e.g., 3,358,169 vs. 3358169c).

## Results

### Phenotyping

Sterile, partly fertile, and fertile genotypes of the BC1F1: S305P/00 × [RIL F7(S64/04/01): S305N/00 × SO37R/05] population were represented by 80, 6, and 59 RILs, respectively. Phenotypic data were not available for 30 of the 175 lines.

Based on phenotypic data, the fertility trait did not follow a normal distribution, as indicated by the Kolmogorov-Smirnov test (*D* = 0.841; *p* < 0.0001; *α* = 0.05).

The whole population was divided into two phenotypic classes: sterile (lines with 1–3 score) and fertile (the remainder of the population). The sterile-to-fertile ratio was 80:65. Chi-square adjustment tests revealed that the population deviated significantly from the expected 1:1 segregation ratio (*χ*^*2*^ = 10,337, *p* < 0.01 at *α* < 0.05).

### Genetic map

**Fig. 1:**
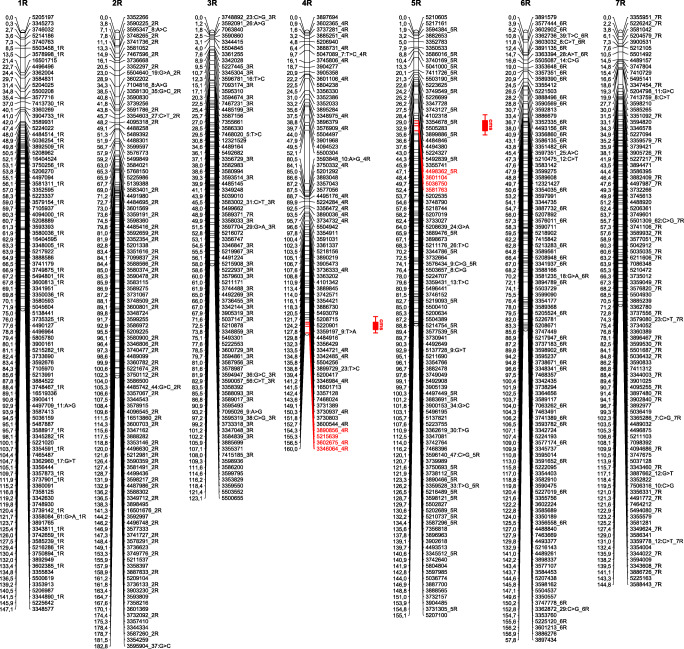
Genetic map of rye based on the RIL S64/04/01 mapping population. Red boxes indicate genomic regions determining male fertility restoration QTLs conferring pollen fertility. The markers closely linked to the pollen fertility restoration loci are indicated in red

Genotyping of the RIL S64/04/01 mapping population produced approximately 36,000 SNP and 128,000 silicoDArT markers. The constructed map consisted of seven LGs (Suppl. File S2, Fig. 1) with 15,516 markers: 643 skeletons, 2418 redundant, and 12,455 added (Table 1). The most extended linkage group was constructed for chromosome 2R, which spanned 182.8 cm and contained 103 markers with one marker per 1.77 cm on average. The shortest LGs were for the 1R (147.1 cm) and 7R (144.7 cm) chromosomes, with 97 and 93 markers, respectively. In total, the map spanned 1070.5 cm with one skeleton marker per 1.66 cm, on average. Despite the high saturation of the map, some gaps between skeleton markers remained (Fig. 1). The most substantial gap, spanning over 28 cm, was identified on the 4R chromosome. The linkage group S-L orientation was based on the known position of markers on the winter rye inbred line Lo7 genetic map (Bauer et al. 2017). Collinearity assessment of RIL S64/04/01 and the map published by Bauer et al. (2017) showed that correlation indices (*p* < 0.0001) were in the range 0.822–0.993 (Table 1), with the purest and best correlations for the 2R and 6R chromosomes, respectively.

**Table 1:** Characteristics of the RIL S64/04/01 genetic linkage map based on silicoDArT and SNP markers

	Chromosome	1R	2R	3R	4R	5R	6R	7R	Total	Average
No. of markers	Skeleton	97	103	79	71	98	102	93	643	92
	Redundant	424	367	258	281	327	410	351	2418	345
	Added	1767	2012	2057	1430	1652	1914	1623	12,455	1779
	All	2288	2482	2394	1782	2077	2426	2067	15,516	2216
Map characteristics	Map length (cm)	147.1	182.8	123.0	160.0	155.1	157.8	144.7	1070.5	152.9
	Map density (cm)	1.52	1.77	1.56	2.25	1.58	1.55	1.56	–	1.66
	Max gap (cm)	7.90	5.84	4.14	28.41	6.75	8.06	7.84	68.94	9.84
	Correlation coefficients of the LGs for RIL F7 and Lo7 maps	0.975	0.822	0.986	0.968	0.931	0.993	0.975	–	–

### Detection of QTLs for pollen fertility restoration

Nonparametric Kruskal-Wallis (K-W) analysis of the RIL S64/04/01 population detected 71 skeleton markers significantly (*p* ≤ 0.05) associated with the fertility trait. An important QTL region controlling fertility restoration was detected on the distal part of the long arm of the 4R chromosome. Markers 3731389, 3730937, 3730803, 3600544, 3890856, 5215639, 3602675, and 3346064, located on the 4R map between 149.0 cm and 160 cm, were evaluated as the most significant (*p* ≤ 0.0001) (Table 2). The association values (K*) for these markers were greater than 38.66. Eleven added markers with *p* values ≤ 0.005 and ≤ 0.001 were also found on 4R, between 129.8 cm and 143.6 cm. Three additional loci were identified on the 3R, 5R, and 7R chromosomes (Table 2). The remaining markers, with a *p* value ≤ 0.01, were dispersed throughout all chromosomes.

**Table 2:** Kruskal-Wallis test showing the association between markers and fertility restoration in rye with CMS Pampa. Only markers with significance values *p* ≤ 0.01 are shown

Chromosome	Marker position (cm)	Marker name	*df*^1^	K_max_^2^	Significance^3^
1R	61.29	3593393S	1	7.67	***
2R	105.27	4485742_44:G > C	1	6.68	***
3R	123.05	5500655	1	8.25	****
4R	129.81	4484917	1	11.85	*****
4R	132.3	3356420	1	8.13	****
4R	133.52	3344722	1	9.45	****
4R	134.81	3342486	1	12.28	****** *
4R	135.76	5504257	1	12.99	******
4R	138.78	3899720_23:T > C	1	9.51	****
4R	139.41	5200418	1	11.49	*****
4R	141.22	3346985	1	14.60	******
4R	141.52	16501714	1	13.36	******
4R	142.42	3357129	1	14.52	******
4R	143.62	7468025	1	13.27	******
4R	149.08	3731389	1	38.66	*******
4R	150.32	3730937	1	45.96	*******
4R	151.82	3730803	1	46.25	*******
4R	152.72	3600544	1	54.94	*******
4R	154.29	3890856	1	63.36	*******
4R	155.25	5215639	1	72.66	*******
4R	156.51	3602675	1	80.71	*******
4R	160.01	3346064	1	63.51	*******
5R	48.38	3601104	1	8.60	****
6R	26.52	5221503	1	7.81	***
7R	70.51	3885239	1	8.13	****
7R	71.15	3362780	1	8.78	****
7R	72.79	3737556	1	6.78	***

^1^*df* degrees of freedom

^2^K_max_ maximum value of the K statistic within the interval

^3^Significance levels: ***p ≤ 0.01, ****p ≤ 0.005, *****p ≤ 0.001, ******p ≤ 0.005, *******p ≤ 0.0001

**Fig. 2:**
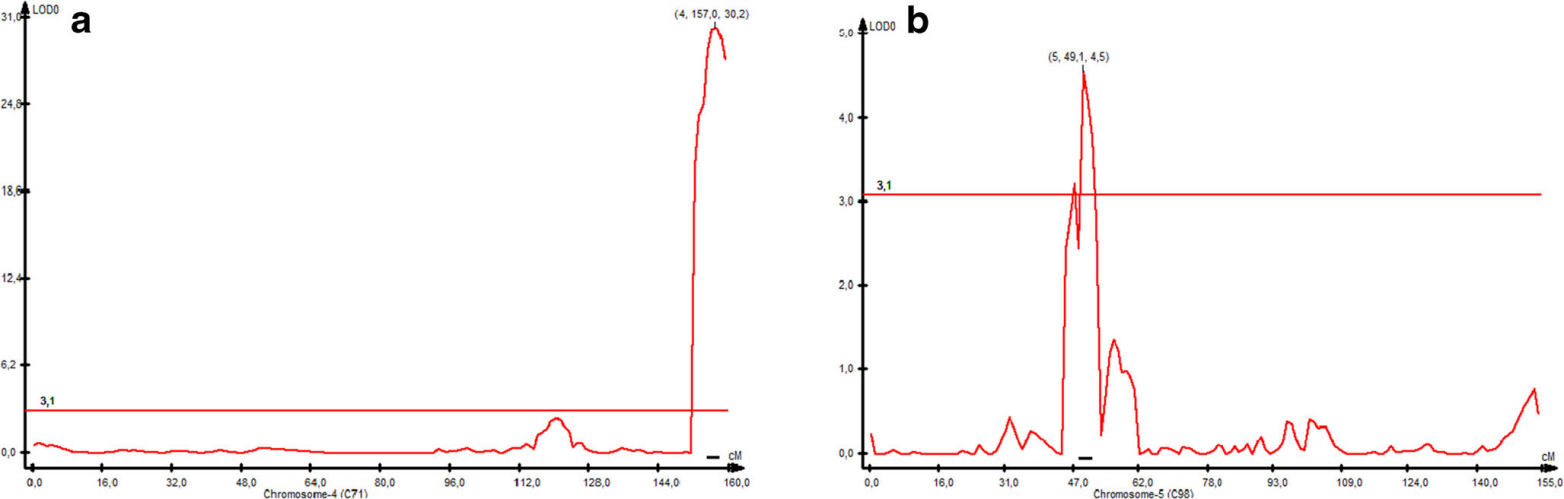
Composite interval mapping demonstrating the position of the QTLs identified on the 4R (**a**) and 5R (**b**) chromosomes based on the RIL S64/04/01 mapping population

Composite interval mapping (CIM) was generally congruent with K-W analysis and confirmed the presence of two QTLs conferring fertility restoration in rye with CMS Pampa. A highly significant QTL (*QRft-4R*) with logarithm of the odds (LOD) score 30.3 (*p* = 1000; LOD = 3.1) was mapped to the distal part of the long arm of the 4R chromosome (Figs. 1 and 2, Table 3) and spanned over 8 cm. *QRft-4R* exhibited additive effects (A = 3.19) and explained up to 60.0% of the phenotypic variance for pollen fertility restoration.

**Table 3:** Characteristics of pollen fertility restoration QTLs identified by CIM based on the RIL S64/04/01 mapping population. LOD: the logarithm of odds; *R*^*2*^ (%): the percentage of phenotypic variance explained by the given QTL; A is the value of the additive effect of the SO37R/05 allele; RecL and RecR indicate the recombination value of the markers in the nearest vicinity of the QTL LOD function maximum

Chr	QTL	Flanking markers (position in cm)	LOD value maximum	LOD maximum position (cm)	A	*R*^*2*^ (%)	RecL	RecR
4R	QRft-4R	5,605,675 (156.51)–5,546,064 (160.01)	30.29	157.00	3.19	60.0	0.009	0.029
5R	QRft-5R	3,601,104(48.38)–5,036,750 (49.73)	4.55	49.01	1.31	5.5	0.000	0.029

*LOD*: the logarithm of odds; *R*^*2*^ (%): the percentage of phenotypic variance explained by the given QTL; A is the value of the additive effect of the SO37R/05 allele; RecL and RecR indicate the recombination value of the markers in the nearest vicinity of the QTL LOD function maximum

The silicoDArT markers flanking *QRft-4R* were located 0.49 cm (3602675) and 3.0 cm (3346064) apart from the LOD maximum (Table 4). The other two closely linked markers were mapped at a distance of 1.75 cm (5215639) and 2.71 cm (3890856). Besides, the QTL region was saturated with six redundant (Table 4) and 202 added markers.

**Table 4:** Arrangements of skeleton markers closely linked to *QRft-4R* and *QRft-5R* in the RIL S64/04/01 mapping population

QTL	Marker	Distance from the LOD maximum (cm)	Redundant markers
QRft-4R	3602675	0.49	5213529, 3575913, 3591086_11:A > G, 4099882, 3595971_25:C > T
QRft-4R	5215639	1.75	–
QRft-4R	3890856	2.71	3899669
QRft-4R	3346064	3.01	–
QRft-5R	3601104	0.63	3,736,545
QRft-5R	5036750	0.72	3903468
QRft-5R	4498362	1.91	4494701, 3357941, 5504657, 3343066, 3341963, 3342917, 3347004, 3342019

The second QTL (*QRft-5R*) was mapped to the 5R chromosome and had a LOD maximum value equal to 4.5 and spanned about 5 cm (Table 3). *QRft-5R* passed the permutation test (LOD 3.1). The maximum LOD value for *QRft-5R* occurred at position 49.0 cm of the map. The 3601104 (0.63 cm), 5036750 (0.72 cm), and 4498362 (1.91 cm) markers were the closest silicoDArTs to the QTL LOD function maximum (Table 4). The QTL region was represented by ten redundant and 52 added markers. The *QRft-5R* was characterized by an additive effect A = 1.31 (Table 3).

### Association mapping

Principal components analysis (PCA) failed to identify any signs of population structure (not shown). A General Linear Model (GLM) approach allowed the identification of 176 SNP and silicoDArT markers associated with the pollen fertility trait at *p* < 0.001. Thirty-seven of those markers had 650 redundant counterparts, increasing the total number of associated markers to 826. The association coefficients (*R*^*2*^) of the markers that passed the Bonferroni test (*p* = 6.14E-07) varied from 0.162 to 0.583. Markers mapped to chromosome 4R. Of the 30 markers with the highest association values, 21 mapped within the *QRft-4R* region (Table 5). The 5605675 marker, with the highest value of *R*^*2*^ (0.583, *p* = 2.21E-28), was 0.49 cm from the LOD function maximum representing *QRft-4R*. None of the associated markers were assigned to the *QRft-5R* region.

**Table 5:** Association mapping results for 30 markers with the highest *p* values

Marker name	Chr	*p* value	*R*^*2*^	Marker position (cm) on the RIL:S64/04/01 genetic map
3602675	4R	2.21E-28	0.583	156.51S
3746061	4R	4.60E-26	0.558	156.51A
3593608	4R	8.85E-26	0.557	156.51A
3579773	4R	1.01E-24	0.582	n.m.
5215639	4R	1.52E-24	0.542	155.25S
3733750	4R	5.50E-24	0.521	156.51A
3746249	4R	8.74E-24	0.531	n.m.
5211969	4R	1.31E-22	0.499	n.m.
7463634	4R	1.42E-22	0.498	156.51A
3582094	4R	2.41E-22	0.500	156.51A
5034752	4R	3.56E-22	0.492	160.01A
3587751	4R	4.19E-22	0.491	156.51A
3341901	4R	3.90E-21	0.472	154.29A
3890856	4R	7.88E-21	0.478	154.29S
3346064	4R	8.92E-21	0.470	160.01S
3589436	4R	2.36E-20	0.480	155.25A
3590835	n.a.	2.37E-20	0.478	n.m.
5203055	n.a.	4.21E-20	0.489	n.m.
7500216	4R	6.66E-20	0.460	156.51A
3743168	4R	9.74E-20	0.467	155.25A
3362765_16:T > A	n.a.	1.81E-19	0.459	n.m.
16404848	4R	6.97E-19	0.443	154.29A
3744939	4R	7.20E-19	0.433	154.29A
3586479	4R	8.14E-19	0.447	154.29A
3589890	n.a.	8.87E-19	0.439	n.m.
3590786	4R	1.15E-18	0.425	154.29A
16520815	4R	1.23E-18	0.441	154.29A
3593839	4R	1.72E-18	0.428	n.m.
3351844	4R	2.08E-18	0.432	155.25A
3580697	n.a.	7.32E-18	0.438	n.m.

*n.a.* not assigned, *n.l.* not mapped

Letters A and S indicate added and skeleton markers, respectively. *R*^*2*^ represents association coefficient, and *p* value indicates the marker trait association probability. Chr represents the chromosome to which the marker is assigned on the RIL S64/04/01 genetic map

### Marker sequence homology

Of the 435 DArTseq/silicoDArT marker sequences, 77.5% sequences were not available in any database sequences. The remaining marker sequences showed similarity to genomic sequences from *Triticum aestivum*, *Hordeum vulgare*, *Aegilops tauschii*, *Oryza sativa*, and *Brachypodium distachyon* (Suppl. Tab. S1)*.* Four of the marker sequences (3887543, 16404848, 3599981, 5500712) approximated to the 4R linkage group in proximity to the 5215639 and 3890856 markers closely linked to *QRft-4R* (1.75 and 2.71 cm, respectively) and exhibited sequence similarity to *Rfm1* (I% = 84, 89, 80, and 90.5, respectively), which is responsible for pollen fertility restoration in barley. Three of the markers (3887543, 16404848, and 5500712) were associated with the fertility trait (*R*^*2*^ = 0.578, 0.473, and 0.443, respectively). The SNP 3362765_16: T > A marker associated with pollen fertility restoration (*R*^*2*^ = 0.459) also shared similarity with the *Rfm1* locus (I% = 100; Suppl. Tab. S1). Marker 7500216 mapped in the vicinity of the 3602675 marker, which was located 0.49 cm from the *QRft-4R* LOD function maximum and was associated with the fertility trait (*R*^*2*^ = 0.460) and had low sequence similarity (I% = 87.9) to the DNA sequence of the fertility restoration gene *Rf1* from *Aegilops tauschii*.

DNA sequence similarities between markers associated/linked with/to the trait and *Rfm1* sequences ranged from 1.00E-04 (16404848) to 1.00E-17 (3362765_16:T > A) (Suppl. Tab. S1). Sequence identity between markers and one of the three rye contigs, Lo7_v2_contig_237103 (1127 bp), Lo7_v2_contig_267616 (4543 bp), and Lo7_v2_contig_80366 (3760 bp), was close to 100% (Bauer et al. 2017). These contigs matched three *Rfm1* gene regions (5177–8797 bp, 452,246–452,967 bp, and 76,142–76,384 bp) with high probability (*E* value = 0.0, 177, and 47, respectively).

Four of the markers (3351619, 3357230, 3358064, 3885888) exhibited similarity to the sequences of the mitochondrial transcription termination factor family (mTERF) gene from *Aegilops tauschii* and also matched one of the three rye Lo7 contigs (Lo7_v2_contig_378957, Lo7_v2_contig_1373077, Lo7_v2_contig_149174) (Bauer et al. 2017). The contigs completely matched the mTERF15 gene sequence (*E* value = 0.0).

Seven markers (7468019, 3358169, 3590786, 3746061, 5037479, 3348274, 4096992) exhibited similarity to the DNA sequence keratin-associated proteins (KAP) 5–4-like and 5–5-like from *Aegilops tauschii.* Marker similarities to rye contigs were 73–100%. Only Lo7_v2_contig_419182 exhibited significant (*E* value = 2E-103) similarity to the 411 bp sequence of keratin-associated 5–5-like protein.

The markers that exhibited similarity to mTERF15 and KAP5–5 were mapped in the vicinity of *QRft-4R* (Suppl. Tab. S1). These markers also showed high association values with fertility restoration, equal to 0.583 and 0.425 for markers with sequence similarities to mTERFs and KAPs, respectively.

Additional annotated genes corresponding to marker sequences flanking both *QRft-4R* and *QRft-5R* are listed in Supplementary Table S1. Among these, the highest *E* values for sequence similarities were obtained for 3599685 (4R), 5212120_43: A > G (5R), 3342019 (5R), and 4092866 (5R) with probable methyltransferase PMT17 (3.00E-25), probable aldo-keto reductase 2 (1.00E-24), putative disease resistance RPP13-like protein 3 (1.00E-24), and serine/threonine-protein phosphatase 2A 57 kDa regulatory subunit B′ (8.00E-26), respectively.

### Marker conversion assays

Of the 35 markers initially selected for conversion, 27 marker sequences were extended based on marker homology to DNA sequences deposited in DNA databases. The extended sequences allowed the development of ten PCR-based markers with polymorphic signals when tested on RILs carrying sterile and fertile QTLs. Of these, five markers localized in the *QRft-4R* region (3602675c, 3575914c, 4099883c, 3358169c, 5500712c) exhibited identical segregation patterns as their unconverted counterparts (Suppl. File S1). Minor differences in marker segregation reflected missing data in the case of some RILs genotyped with DArTseq markers. Marker 3602675 and its redundant counterparts (3575914, 4099883) mapped at 156.51 cm on the 4R chromosome. These markers are 0.49 cm from the *QRft-4R* LOD maximum value. Markers 3358169 and 5500712 revealed sequence similarities with (KAP) 5–4-like and *Rfm1*, respectively. Marker 3358169 approximated to the 4R map at the 152.72 cm position, and marker 5500712 was at 155.25 cm. Of the 23 fertile RILs of the S64/04/01 mapping population, the markers were present in 19 lines. None of the markers were identified in any of the sterile plants. For the three partly fertile RILs, two segregated as fertile and one as sterile RILs.

## Discussion

Phenotypic assessment of individuals of the BC1F1 population divided progeny into two main phenotypic classes: male sterile and male fertile. Partially fertile plants were also present in a low number (6 plants: 5–6 score according to the bonitation scale) and, due to the presence of pollen, were included into the fertile class in the segregation analysis. A similar segregation of male fertile and male sterile plants was recently reported for rye populations with C and Pampa cytoplasms, where the presence of a major *Rf* gene on the 4R chromosome was documented (Stojałowski et al. 2011; Stracke et al. 2003). The ratio of the phenotypic classes in the studied hybrids deviated significantly from the 1:1 segregation ratio typical of the monogenic model of inheritance in a RIL population. The observed data may be explained in several ways: (1) The distortion is due to the lack of phenotypic data of the 30 missing genotypes; (2) phenotyping was performed in a single environment without repeats; and (3) the analyzed population has several QTLs conferring pollen fertility restoration traits in the rye. Although 30 of the 175 cases were not phenotyped, the dataset available from the remaining 145 lines is reasonably large. Thus, missing cases should not significantly affect the segregating ratio. Pollen fertility restoration in rye with CMS Pampa is usually only minimally affected by environmental conditions, at least for the main QTLs (Geiger et al. 1995). As the vast majority of the BC1F1 plants were sterile or fertile, with only a few partially fertile plants, further assessment of phenotype under different environments would be unlikely to affect segregation and was therefore not performed (Geiger et al. 1995). Furthermore, the trait was clearly expressed, suggesting that at least one major QTL was represented in the RIL7 population and that other QTLs were either of minor importance or were modifying genes that were previously reported in rye (Miedaner et al. 2000).

Mapping of agronomically essential traits requires genetic maps with a high density of markers (Cockram and Mackay, 2018). To date, all the mapping populations dedicated to studies of pollen fertility restoration in rye utilized F2 progeny (Miedaner et al., 2000; Stracke et al. 2003; Hackauf et al. 2012; Stojałowski et al. 2017). However, the frequency of polymorphic DArT, silicoDArT, and SNP markers in a rye F2 population (544 × Ot0–20 BC5F2) were 9.3, 8, and 4.6%, respectively, whereas frequencies in a RIL-S population (generation F5) were 19.6, 58.7, and 29.7% (Milczarski et al. 2016). Moreover, RILs better support map resolution due to recombination frequency accumulation during each generative cycle (Xu et al. 2017), and they are immortal populations (Cockram and Mackay, 2018). Thus, the employment of recombinant inbred lines is preferred over F2 populations (Cockram and Mackay, 2018). However, the development of RILs is time-consuming and can be challenging in some crops, like rye, due to inbreeding depression (Singh and Singh, 2015).

In this study, a specially designed RIL-based mapping population consisting of 175 lines on non-sterilizing cytoplasm, but carrying pollen fertility restoration QTLs that originated from contrasting parental lines, was evaluated and exploited for genetic map construction. The final map length was 1070.5 cm, which was 174 cm and 533 cm shorter than the map of rye inbred line Lo7 (Bauer et al. 2017) and the consensus map of five RIL-based mapping populations (Milczarski et al. 2011), respectively. Chromosome lengths ranged from 139.9 cm (7R) to 214.5 cm (5R) and, in case of chromosomes 1R, 2R and 7R, were similar to the lengths of those constructed for Lo7 (Bauer et al. 2017). However, on average, chromosome lengths differed by 25% for 3R, 4R, and 5R. The results presented by Milczarski et al. (2011) showed that the origin of the population strongly influenced the length of individual chromosomes, which differed by up to 220 cm (5R) when the same DArT technology was used for genotyping of five RIL populations originating from nine parental lines. The average map density of RIL S64/04/01 was 1.66 cm, within the 1.1–2.75 cm range described for DArTs in the case of other rye RIL-based populations (Bolibok-Brągoszewska et al. 2009; Milczarski et al. 2011). Somewhat higher map density (0.47 cm) was reported by Milczarski et al. (2016), who succeeded in mapping as many as 2448 silicoDArT and SNP unique loci and 928 DArT markers using 92 individuals of the RIL-S (F5) population. The difference in map density results from the fact that the map density of the RIL7 map was estimated based on highly “stable” (minimum missing and best segregation ratio) skeleton markers (without redundant and added markers). To our knowledge, the RIL7 based genetic map presented here is the first to be dedicated to studies of pollen fertility restoration in rye with CMS Pampa.

As the analyzed fertility trait failed to have a normal distribution, nonparametric Kruskal-Wallis (K-W) analysis was employed for the detection of QTLs (Myśków et al. 2014; Stojałowski et al. 2017). Four genomic regions that mapped to the 4R, 3R, 5R, and 7R chromosomes were detected. Composite interval mapping confirmed the presence of a single highly significant QTL on the long arm of the 4R chromosome and a minor QTL on the 5R chromosome. The major QTL on 4R explained 60% of the variance of fertility restoration, comparable to IRAN IX (68%) and Pico Gentario (59%) based materials (Miedaner et al. 2000).

It is rare for European breeding materials to carry such a strong QTL on chromosome 4R (Miedaner et al. 2000). The QTL was probably introduced to a pollen donor (SO37R/05) of the RIL S64/04/01 population from Iranian or Argentinian sources. The identified region is congruent with earlier reports evaluating Iranian primitive rye populations IRAN IX and Altevogt 14,160, Argentinian landrace Pico Gentario, and European line L18 (Miedaner et al. 2000; Hackauf et al. 2017). Interestingly, the major QTL location is also congruent with studies on pollen fertility restorers in the case of CMS C (Stojałowski et al. 2005) and G (Börner et al. 1998) in rye. However, it is not clear whether the same gene is responsible for pollen fertility restoration in all types of sterilizing cytoplasms.

A second QTL of minor importance was identified on chromosome 5R and explained 5.5% of the variance, and this could justify the lack of monogenic segregation of the trait as indicated by phenotypic data. Similar results concerning a QTL on the 5R chromosome were described previously (Miedaner et al. 2000), where a minor locus explained 11% of the phenotypic variation of fertility restoration in the L18 line. Unfortunately, the two QTLs on chromosome 5R cannot be easily compared because different marker systems were used in the two studies.

The association mapping analysis used to identify markers associated with the trait but not necessarily present on the map was congruent with QTL analysis in the case of the major QTL only. In total, four markers tightly linked to the QTL and 176 markers associated with pollen fertility restoration were identified on the long arm of the 4R chromosome.

A comparison of the marker DNA sequences against sequences stored in various online databases at NCBI (Suppl. Tab. S1) was performed for the identification of their functional annotations. Five markers mapped to the 4R QTL and/or associated with fertility restoration exhibited similarity to the *Rfm1* gene sequence mapped to the chromosome 6H in barley (Matsui et al. 2001; Murakami et al. 2005; Rizzolatti et al. 2017). Moreover, the 69 nucleotide-long markers nearly perfectly (99–100% identity) matched the rye Lo7 contigs (Bauer et al. 2017), which exhibited high similarities to the *Rfm1* gene sequence. Synteny-based studies showed that the 6HS chromosome distal region (Martis et al. 2013) carrying the restorer *Rfm1* gene (Matsui et al. 2001; Murakami et al. 2005) was homologous to the rye 4RL where the *Rfp1* and *Rfp3* genes were mapped (Hackauf et al. 2012 and 2017). Analysis of homology between these chromosomal regions and 3S in *Brachypodium*, 4S in sorghum, and 2S in rice revealed that collinearity was maintained among these grass species (Hackauf et al. 2012; Ui et al. 2015). Thus, the rye analog of the *Rfm1* gene is a reasonable candidate for the pollen fertility restoration gene in rye with CMS Pampa. Nevertheless, due to the perfect collinearity observed at the genetic map level between the *Rfm1* locus in barley and *Brachypodium* (Ui et al. 2015), and a small number of rearrangements between the *Rfp3* genomic region in rye and *Brachypodium*, Hackauf et al. (2017) concluded that *Rfp3* and *Rfm1* might represent independent fertility restorer genes. Thus, it is likely that the markers identified in RIL S64/04/01 associate with the *Rfp1* or *Rfp2* gene sequences. The nucleotide sequences of barley *Rfm1*, rye *Rfp1* and *Rfp2*, and a segment of Bd3 in *Brachypodium* that was mapped proximal to *Rfp3* (Hackauf et al. 2017) indicate that the locus carries a tandem repeat of a gene encoding a PLS-DYW-class pentatricopeptide repeat (PPR) protein. A major function of PLS PPR proteins possessing C-terminal domains (E or DYW) is C-to-U RNA editing in plant organelles (Hammani and Giegeè 2014; Small et al. 2019), suggesting a potential role for RNA editing in pollen fertility restoration in rye.

Blasting marker sequences (3351619, 3357230, 3358064, 3885888) against DNA databases indicated that a mitochondrial transcription termination factor family (mTERF) gene identified in a rye segment carrying *Rfp1* and *Rfp3* might also participate in pollen fertility restoration in rye (Hackauf et al. 2012, Hackauf et al. 2017). Recently, a novel restorer locus, *Rfm3*, was found to be closely linked to mTERF in barley (Bernhard et al. 2019). The CMS unstable mother plants, which were homozygous at the *Rfm3* locus, had significantly higher grain setting under elevated temperature until ripening. The results are comparable to those in maize (Zhao et al. 2014) and suggest that mTERF genes are up- and down-regulated depending on their environmental conditions. Thus, in barley, *Rfm3* may be responsible for undesired fertility restoration in CMS mother lines in the absence of the functional *Rfm1* restorer gene (Bernhard et al. 2019). The putative roles of mTERF proteins in the context of fertility restoration in rye have not yet been determined.

Further studies of marker sequence similarities showed that seven markers identified in the study indicated the role of a third gene (keratin-associated protein (KAP) 5–4-like and 5–5-like) which belongs to the KAP type 5 family (Jenkins and Powell 1994). KAP and homologous KAP gene functions in plants are poorly elucidated. Zhou et al. (2009) showed that qPE9–1, a putative homologous gene of KAP 5–4 in humans, regulated rice panicle erectness and played pleiotropic roles in an array of plant architecture and yield traits. The functional resemblance of the protein encoded by the KAP5–4 gene to the *wali6* protein may suggest an involvement to drought resistance (Yang et al. 2012). The presence of the linked markers within the *QRft-4R* region and strong marker associations with the trait may suggest that keratin could be essential for pollen fertility restoration. However, the similarity of the DArTseq sequence markers and KAPs DNA sequences might be due to a common domain in the structure of the RF2 and keratin proteins. The KAP5 family shows extensive amino acid sequence conservation, and all the proteins are composed almost entirely of cysteine-rich and glycine-rich repeats (Jenkins and Powell 1994). Map-based cloning demonstrated that *Rf2* in rice encodes a protein comprising a glycine-rich region (GRP) (Itabashi et al. 2011) that is probably responsible for direct interaction with the CMS-causing protein or which may cooperate with other proteins via the glycine-rich region to form a multi-molecular complex participating in fertility restoration (Itabashi et al. 2011). A further study in rice (Hu et al. 2012) showed that the *Rf5* gene encodes a PPR protein that interacts with a glycine-rich domain protein GRP162 to bind to atp6-orfH79 and build restoration of the RFC fertility complex in Hong-Lian CMS lines. Thus, it is possible that the *QRft-4R* region detected in rye contains several relevant genes, including PPR, mTERF, and GRP proteins.

The identified markers that were linked to or associated with fertility restoration in rye and exhibited similarities to putative pollen fertility genes were converted to sequence-specific PCR conditions to facilitate their use in marker-assisted programs. Conversion efficiency can depend on the type of maker. For example, conversion of RAPD markers is relatively inefficient due to the lack of sequence uniformity of bands forming a marker and the involvement of many practical steps including cloning and sequencing (Mikolajczyk et al., 2008). An added complexity is that not all primers designed for amplification are capable of amplifying expected polymorphisms (Xie et al. 2008, Lee et al. 2010). This is somewhat alleviated when marker sequences derived via NGS are available (Macko-Podgórni et al. 2014; Fiust et al. 2015; Niedziela et al., 2015). However, as only relatively short sequences are generated, their direct conversion (i.e., into sequence-specific ligation amplification markers) is not practical (Milczarski et al., 2016). Analysis of DArTseq/silicoDArT marker sequence similarities allows sequences to be extended, and these longer sequences can be utilized for the development of PCR-based markers for MAS purposes. The efficiency of such conversion can reach 100% and usually 50–60% of these are polymorphic (Niedziela et al., 2015; Fiust et al., 2015). In this study ten of 27 markers were successfully converted. However, only five markers (3602675, 3575914, 4099883, 3358169, 5500712) present within the *QRfp-4R* region resulted in amplifications that followed expected segregation based on 48 RILs chosen from the S64/04/01 mapping population. One of the tested markers (5500712) revealed sequence similarities to *Rfm1*, which was identified previously in *Hordeum vulgare* (Matsui 2001). Although the markers were located in different positions within *the QRft-4R* region (3602675, 3575914, 4099883: 156.51 cm; 3,358,169: 152.72 cm; 5,500,712: 155.25 cm), their segregation patterns were identical. For MAS purposes, the markers will be tested on a differentiated pool of rye genotypes.

In this study, a QTL located on the 4R chromosome was confirmed as responsible for efficient fertility restoration in rye with CMS Pampa cytoplasm. A set of silicoDArT and SNP markers linked with the *QRfp-4R* region was identified for the first time. The presence of *Rfp* and mTERF genes within *QRfp-4R* was proved based on the sequence homology approach. Five novel markers with practical utility were obtained by conversion of silicoDArTs to single-marker assay formats. Moreover, a QTL with minor effects on fertility was identified on chromosome 5R.

## Supplementary Information

ESM 1 (DOCX 38 kb)

ESM 2 (XLSX 20 kb)

ESM 3 (XLSX 23 kb)

## References

[CR1] Bauer E, Schmutzer T, Barilar I, Mascher M, Gundlach H, Martis MM, Twardziok SO, Hackauf B, Gordillo A, Wilde P, Schmidt M, Korzu V, Mayer KFX, Schmid K, Schön C-C, Scholz U (2017). Towards a whole-genome sequence for rye (*Secale cereale* L). The Plant Journal.

[CR2] Bednarek PT, Masojć P, Lewandowska R, Myśków B (2003). Saturating rye genomic map with amplified fragment length plymorphism (AFLP) and random amplified polymorphic DNA (RAPD) markers. J Appl Genet.

[CR3] Bernhard T, Koch M, Snowdon RJ, Friedt W, Wittkop B (2019). Undesired fertility restoration in msm1 barley associates with two mTERF genes. Theor Appl Genet.

[CR4] Bolibok-Brągoszewska H, Heller-Uszyńska K, Wenzl P, Uszyński G, Kilian A, Rakoczy-Trojanowska M (2009). DArT markers for the rye genome - genetic diversity and mapping. BMC Genomics.

[CR5] Börner A, Korzun V, Polley A, Malyshev S, Melz G (1998). Genetics and molecular mapping of a male-fertility restoration locus (*Rfg1*) in rye (*Secale cereale* L.). Theor Appl Genet.

[CR6] Bradbury PJ, Zhang DE, Kroon TM, Casstevens Y, Ramdoss Y, Buckler ES (2007). TASSEL: software for association mapping of complex traits in diverse samples. Bioinformatics.

[CR7] Brown GG, Formanová N, Jin H, Wargachuk R, Dendy C, Patil P, Laforest M, Zhang J, Cheung WY, Landry BS (2003). The radish Rfo restorer gene of Ogura cytoplasmic male sterility encodes a protein with multiple pentatricopeptide repeats. Plant J.

[CR8] Bushuk W (2001). Rye production and uses worldwide. Cereal Foods World.

[CR9] Cockram J, Mackay I (2018). Genetic mapping populations for conducting high-resolution trait mapping in plants. Adv Biochem Eng Biotechnol.

[CR10] Desloire S, Gherbi H, Laloui W, Marhadour S, Clouet V, Cattolico L, Falentin C, Giancola S, Renard M, Budar F, Small I, Caboche M, Delourme R, Bendahmane A (2003). Identification of the fertility restoration locus, Rfo, in radish, as a member of the pentatricopeptide-repeat protein family. EMBO Rep.

[CR11] Fiust A, Rapacz M, Wójcik-Jagła M, Tyrka M (2015). Development of DArT-based PCR markers for selecting drought-tolerant spring barley. J Appl Genet.

[CR12] Frisch M, Melchinger AE (2000). The length of the intact donor chromosome segment around a target gene in marker-assisted backcrossing. Genetics.

[CR13] Geiger HH, Miedaner T (1996). Genetic basis and phenotypic stability of male-fertility restoration in rye. Vortr Pflanzenzüchtg.

[CR14] Geiger HH, Morgenstern K (1975). Angewandt-genetische Studien zur cytoplasmatischen Pollensterilität bei Winterroggen. Theor Appl Genet.

[CR15] Geiger HH, Schnell FW (1970). Cytoplasmic male sterility in rye (*Secale cereale* L.). Crop Sci.

[CR16] Geiger HH, Yuan Y, Miedaner T, Wilde P (1995). Environmental sensitivity of cytoplasmic genic male sterility (CMS) in Secale cereale L. in: Kück U and Wricke G (eds), genetic mechanisms for hybrid breeding. Adv Plant Breed.

[CR17] Hackauf B, Bauer E, Korzun V, Miedaner T (2017). Fine mapping of the restorer gene Rfp3 from an Iranian primitive rye (*Secale cereale* L.). Theor Appl Genet.

[CR18] Hackauf B, Korzun V, Wortmann H, Wilde P, Wehling P (2012). Development of conserved ortholog set markers linked to the restorer gene Rfp1 in rye. Mol Breed.

[CR19] Hackauf B, Wehling P (2003). Development of microsatellite markers in rye: map construction. Plant Breed Seed Sci.

[CR20] Hammani K, Giegeè P (2014) RNA metabolism in plant mitochondria. Trends plant Sci. 19 380-389 10.1016/j.tplants.2013.12.008 24462302

[CR21] Hammer Ø, Harper DAT, Ryan PD (2001). PAST: paleontological statistics software package for education and data analysis. Palaeontol Electron.

[CR22] Hu J, Wang K, Huang W, Liu G, Gao Y, Wang J, Huang Q, Ji Y, Qin X, Wan L, Zhu R, Li S, Yang D, Zhua Y (2012). The rice pentatricopeptide repeat protein RF5 restores fertility in hong-lian cytoplasmic male-sterile lines via a complex with the glycine-rich protein GRP162. Plant Cell.

[CR23] Itabashi E, Iwata N, Fujii S, Kazama T, Toriyama K (2011) The fertility restorer gene, Rf2, for Lead Rice-type cytoplasmic male sterility of rice encodes a mitochondrial glycine-rich protein. The Plant Journal 65(3):359–367 10.1111/j.1365-313X.2010.04427.x 21265890

[CR24] Jenkins BJ, Powell BC (1994). Differential expression of genes encoding a cysteine-rich keratin family in the hair cuticle. J Invest Dermatol.

[CR25] Kazama T, Nakamura T, Watanabe M, Sugita M, Toriyama K (2008). Suppression mechanism of mitochondrial ORF79 accumulation by Rf1 protein in BT-type cytoplasmic male sterile rice. Plant J.

[CR26] Kazama T, Toriyama K (2003). A pentatricopeptide repeat-containing gene that promotes the processing of aberrant atp6 RNA of cytoplasmic male-sterile rice. FEBS Lett.

[CR27] Khlestkina EK, Than MHM, Pestsova EG, Röder MS, Malyshev SV, Korzun V, Börner A (2004). Mapping of 99 new microsatellitederived loci in rye (*Secale cereale* L.) including 39 expressed sequence tags. Theor Appl Genet.

[CR28] Klein RR, Klein PE, Mullet JE, Minx P, Rooney WL, Schertz KF (2006). Fertility restorer locus Rf1 of sorghum (Sorghum bicolor L.) encodes a pentatricopeptide repeat protein not present in the colinear region of rice chromosome 12. Theor Appl Genet.

[CR29] Kobyljanskij VD (1969). About genetics of cytoplasmic male sterility in winter rye (K genetike citoplazmatičeskoj mužskoj steril’nosti u ozimoj rži). Genet Moskva.

[CR30] Korzun V, Malyshev S, Kartel N, Westermann T, Weber WE, Börner A (1998). A genetic linkage map of rye (*Secale cereal* L.). Theor Appl Genet.

[CR31] Łapiński M, Stojałowski S (2003). Occurrence and genetic identity of male sterility-inducing cytoplasm in rye (*Secale* spp.). Plant Breed Seed Sci.

[CR32] Lee J, Yoon JB, Han J-H, Lee WP, Kim SH, Park HG (2010). Three AFLP markers tightly linked to the genic male sterility ms3 gene in chili pepper (Capsicum annuum L.) and conversion to a CAPS marker. Euphytica.

[CR33] Lehmann EL (1975). Nonparametrics: statistical methods based on ranks.

[CR34] Ma XF, Wanous MK, Houchins K, Rodriguez Milla MA, Goicoechea PG, Wang Z, Xie M, Gustafson JP (2001). Molecular linkage mapping in rye (*Secale cereale* L). Theor Appl Genet.

[CR35] Macko-Podgórni A, Iorizzo M, Smółka K, Simon PW, Grzebelus D (2014). Conversion of a diversity arrays technology marker differentiating wild and cultivated carrots to a co-dominant cleaved amplified polymorphic site marker. Acta Biochim Pol.

[CR36] Martis MM, Zhou R, Haseneyer G, Schmutzer T, Vrána J, Kubaláková M, König S, Kugler KG, Scholz U, Hackauf B, Korzun V, Schön C-C, Doležel J, Bauer E, Mayer KFX, Stein N (2013). Reticulate evolution of the rye genome. Plant Cell.

[CR37] Masojć P, Myśków B, Milczarski P (2001). Extending a RFLP-based genetic map of rye using random amplified polymorphic DNA (RAPD) and isozyme markers. Theor Appl Genet.

[CR38] Matsui K (2001). Molecular mapping of a fertility restorationlocus (Rfm1) for cytoplasmic male sterility in barley (*Hordeum vulgare* L.). Theor Appl Genet.

[CR39] Melz G, Adolf K (1991). Genetic analysis of rye (*Secale cereal* L.). genetics of male sterility of the G-type. Theor Appl Genet.

[CR40] Melz Gi, Melz Gu, Hartmann F (2001) Genetics of a malesterile rye of “G-type” with results of the first F1 hybrids. In proc. Int. Symp. on Rye Breed. and Gen. EUCARPIA, Radzikow, pp 43–50

[CR41] Miedaner T, Geiger HH (2015). Biology, genetics, and management of ergot (*Claviceps* spp.) in rye, sorghum, and pearl millet. Toxins.

[CR42] Miedaner T, Glass C, Dreyer F, Wilde P, Wortmann H, Geiger HH (2000). Mapping of genes for male-fertility restoration in ‘Pampa’ CMS winter rye (*Secale cereale* L.). Theor Appl Genet.

[CR43] Miedaner T, Herter CP, Goßlau H, Wilde P, Hackauf B (2017) Correlated effects of exotic pollen-fertility restorer genes on agronomic and quality traits of hybrid rye. Plant Breed. 10.1111/pbr.12456

[CR44] Miedaner T, Wilde P, Wortmann H (2005). Combining ability of exotic sources for male-fertility restoration in Pampa CMS of hybrid rye. Plant Breed.

[CR45] Mikolajczyk K, Dabert M, Nowakowska J, Podkowinski J, Poplawska W, Bartkowiak-Broda I (2008). Conversion of the RAPD OPC021150 marker of the Rfo restorer gene into a SCAR marker for rapid selection of oilseed rape. Plant Breed.

[CR46] Milczarski P, Banek-Tabor A, Lebiecka K, Stojałowski S, Myśków B, Masojć P (2007). New genetic map of rye composed of PCR-based molecular markers and its alignment with the reference map of the DS2 × RXL10 intercross. J Appl Genet.

[CR47] Milczarski P, Bolibok-Brągoszewska H, Myśków B, Stojałowski S, Heller-Uszyńska K, Góralska M, Brągoszewski P, Uszyński G, Kilian A, Rakoczy-Trojanowska M (2011). A high density consensus map of rye (Secale cereale L.) based on DArT markers. PLoS one.

[CR48] Milczarski P, Hanek M, Tyrka M, Stojałowski S (2016). The application of GBS markers for extending the dense genetic map of rye (*Secale cereale* L.) and the localization of the Rfc1 gene restoring male fertility in plants with the C source of sterility-inducing cytoplasm. J Appl Genet.

[CR49] Myśków B, Hanek M, Banek-Tabor A, Maciorowski R, Stojałowski S (2014). The application of high-density genetic maps of rye for the detection of QTLs controlling morphological traits. J Appl Genet.

[CR50] Niedziela A, Mańkowski D, Bednarek PT (2015). Diversity arrays technology-based PCR markers for marker assisted selection of aluminum tolerance in triticale (x *Triticosecale* Wittmack). Mol Breed.

[CR51] Rizzolatti C, Bury P, Tatara E, Pin PA, Rodde N, Berges H, Budar F, Mireau H, Gielen J (2017) Map-based cloning of the fertility restoration locus Rfm1 in cultivated barley (*Hordeum vulgare*). Euphytica 213-276

[CR52] Ronin Y, Minkov D, Mester D, Akhunov E, Korol A (2015) Building ultra-dense genetic maps in the presence of genotyping errors and missing data. Advances in wheat genetics: from genome to field. Springer, Tokyo, pp 127-133

[CR53] Saal B, Wricke G (1999). Development of simple sequence repeat markers in rye (*Secale cereale* L.). Genome.

[CR54] Saal B, Wricke G (2002). Clustering of amplified fragment length polymorphism markers in a linkage map of rye. Plant Breed.

[CR55] Sánchez-Sevilla JF, Horvath A, Botella MA, Gaston A, Folta K, Kilian A, Denoyes B, Amaya I (2015) Diversity arrays technology (DArT) marker platforms for diversity analysis and linkage mapping in a complex crop, the octoploid cultivated strawberry (*Fragaria* × *Ananassa*) 16;10(12):e0144960 10.1371/journal.pone.0144960 PMC4682937 26675207

[CR56] Singh BD, Singh AK (2015). Mapping populations. Marker-assisted plant breeding: principles and practices.

[CR57] Small ID, Schallenberg-Rüdinger M, Takenaka M, Mireau H, Ostersetzer-Biran O (2019). Plant organellar 436 RNA editing: what 30 years of research has revealed. Plant J.

[CR58] Stojałowski S, Hanek M, Orłowska M, Sobczyk M (2017) DArT markers linked with genes controlling restoration of male fertility in hybrid rye cultivars with improved pollen shedding. Folia Pomer. Univ. Technol. Stetin. Agric. Aliment. Pisc. Zootech. 338(44)4:205-216

[CR59] Stojałowski S, Jaciubek M, Masojć P (2005). Rye SCAR markers for male fertility restoration in the P cytoplasm are also applicable to marker-assisted selection in the C cytoplasm. J Appl Genet.

[CR60] Stojałowski SA, Milczarski P, Hanek M, Bolibok-Brągoszewska H, Myśków B, Kilian A, Rakoczy-Trojanowska M (2011) DArT markers tightly linked with the Rfc1 gene controlling restoration of male fertility in the CMS-C system in cultivated rye (*Secale cereale* L.) J Appl genetics 52:313-318 10.1007/s13353-011-0049-x PMC3132309 21559995

[CR61] Stracke S, Schilling AG, Förster J, Weiss C, Glass C, Miedaner T, Geiger HH (2003). Development of PCR-based markers linked to dominant genes for male-fertility restoration in Pampa CMS of rye (Secale cereale L.). Theor Appl Genet.

[CR62] Ui H, Sameri M, Pourkheirandish M, Chang M-C, Shimada H, Stein N, Komatsuda T, Handa H (2015). High-resolution genetic mapping and physical map construction for the fertility restorer Rfm1 locus in barley. Theor Appl Genet.

[CR63] Uyttewaal M, Arnal N, Quadrado M, Martin-Canadell A, Vrielynck N, Hiard S, Gherbi H, Bendahmane A, Budar F, Mireau H (2008). Characterization of *Raphanus sativus* pentatricopeptide repeat proteins encoded by the fertility restorer locus for ogura cytoplasmic male sterility. Plant Cell.

[CR64] Van Ooijen JW (2004). MapQTL®5, software for the mapping of quantitative trait loci in experimental populations.

[CR65] Voorrips RE (2002). MapChart: software for the graphical presentation of linkage maps and QTLs. The Journal of Heredity.

[CR66] Wang S, Basten CJ, Zeng ZB (2007) Windows QTL cartographer:2.5 http://statgen.ncsu.edu/qtlcart/WQTLCart.htm

[CR67] Wang ZH, Zou YJ, Li XY, Zhang QY, Chen L, Wu H, Su DH, Chen YL, Guo JX, Luo D, Long YM, Zhong Y, Liu YG (2006). Cytoplasmic male sterility of rice with boro II cytoplasm is caused by a cytotoxic peptide and is restored by two related PPR motif genes via distinct modes of mRNA silencing. Plant Cell.

[CR68] Xie YZ, Hong DF, Xu ZH, Liu PW, Yang GS (2008). Identification of AFLP markers linked to the epistatic suppressor gene of a recessive genic male sterility in rapeseed and conversion to SCAR markers. Plant Breed.

[CR69] XlStat (2019) https://www.xlstat.com/en/solutions/pre-mium. p Accessed 10 December 2018

[CR70] Xu Y, Li P, Yang Z, Xu C (2017). Genetic mapping of quantitative trait loci in crops. The Crop Journal.

[CR71] Yang L, Fu F-L, Deng L-Q, Zhou S-F, Yong T-M, Li W-C (2012). Cloning and characterization of functional keratin associated protein 5-4 gene in maize. Afr J Biotechnol.

[CR72] Zhao Y, Cai M, Zhang X, Li Y, Zhang J, Zhao H, Kong F, Zheng Y, Qiu F (2014). Genome-wide identification, evolution and expression analysis of mTERF gene family in maize. PLoS One.

[CR73] Zhou Y, Zhu J, Li Z, Yi C, Liu J, Zhang H, Tang S, Gu M, Liang G (2009). Deletion in a quantitative trait gene qPE9-1 associated with panicle erectness improves plant architecture during rice domestication. Genetics.

