# Supplementary material for: Genetic mapping of pollen fertility restoration QTLs in rye (Secale cereale L.) with CMS Pampa
Source: J Appl Genet. 2021 Jan 7;62(2):185–98. doi: 10.1007/s13353-020-00599-9 (PMC8032618; doi:10.1007/s13353-020-00599-9)
Supplement: Supplementary file 1 — (DOCX 38 kb) [file 13353_2020_599_MOESM1_ESM.docx]

**Supplementary Table S1.** SNP and silicoDArT markers linked to or/and associated with pollen fertility restoration in rye and sharing significant sequence similarity with known genes/proteins. Each of the markers was 69 bases long. Identity percentage: I (%) similarity between the query and subject sequences over the length of the coverage area; Query Cover percentage: QC (%) of the query sequence overlapping the subject sequence; E-value: similarity probability value.

| **Chr** | **Marker name** | **Matched species** | **Homolog** | **QC (%)** | **E-value** | **I (%)** | **Accession number** |
| --- | --- | --- | --- | --- | --- | --- | --- |
| Markers linked to the pollen fertility restoration locus (**S**-skeleton; **R**-redundant; **A**-added) Markers associated with the trait (**AS)** are indicated | | | | | | | |
| 4R | **3890856 (S, AS)** | *Triticum aestivum* | clone: tplb0045f07, cultivar Chinese Spring | 97 | 2.00E-08 | 80.60 | AK451238.1 |
| 4R | 3887543 (A, AS) | *Hordeum vulgare* | Rfm1 gene locus, complete sequence | 72 | 3.00E-06 | 84.00 | MF443757.1 |
| 4R | 16404848 (A, AS) | *Hordeum vulgare* | Rfm1 gene locus, complete sequence | 53 | 1.00E-04 | 89.19 | MF443757.1 |
| 4R | 16404800 (A, AS) | *Triticum aestivum* | mRNA for BEL1-type homeodomain protein | 76 | 3.00E-15 | 94.34 | AB546644.1 |
| 4R | 3351619 (A, AS) | *Aegilops tauschii* | transcription termination factor MTERF15, mitochondrial-like | 68 | 2.00E-06 | 85.11 | XM_020299871.1 |
| 4R | 3599685 (A, AS) | *Aegilops tauschii* | probable methyltransferase PMT17 | 98 | 3.00E-25 | 98.53 | XM_020320230.1 |
| 4R | **3346064 (S, AS)** | *-* | ª n.a. |  |  |  |  |
| 4R | **3602675 (S, AS)** | *-* | n.a. |  |  |  |  |
| 4R | 3361087 (A, AS) | *Aegilops tauschii* | uncharacterized LOC109745189 | 100 | 2.00E-20 | 92.75 | XM_020304321.1 |
| 4R | 3734528 (A, AS) | *Aegilops tauschii* | large proline-rich protein BAG6 | 79 | 2.00E-15 | 94.55 | XM_020293651.1 |
| 4R | 3601994_45:G>C (A, AS) | *Aegilops tauschii* | putative F-box protein At5g42430 | 86 | 1.00E-22 | 100.00 | XM_020329710.1 |
| 4R | 7500216 (A, AS) | *Aegilops tauschii* | PREDICTED: protein Rf1, mitochondrial-like | 47 | 0.018 | 87.88% | XM_020326340.1 |
| 4R | 7468019 (A, AS) | *Aegilops tauschii* | keratin-associated protein 5-5-like | 39 | 0.005 | 96.3% | XM_020293636.1 |
| 4R | **5215639 (S, AS)** | *-* | n.a. |  |  |  |  |
| 4R | 3357230 (A, AS) | *Aegilops tauschii* | transcription termination factor MTERF6, chloroplastic/mitochondrial-like | 57 | 4.00E-07 | 90.00 | XM_020309658.1 |
| 4R | 3358064 (A, AS) | *Aegilops tauschii* | transcription termination factor MTERF15, mitochondrial-like | 66 | 2.00E-10 | 91.30 | XM_020340783.1 |
| 4R | 3358169 (A) | *Aegilops tauschii* | keratin-associated protein 5-4-like | 65 | 9.00E-13 | 97.78 | XM_020293637.1 |
| 4R | 3364878 (A, AS) | *Hordeum vulgare* | mRNA for predicted protein, complete cds, clone: NIASHv3030H04 | 75 | 8.00E-04 | 78.85 | AK373417.1 |
| 4R | 3576343 (A, AS) | *Aegilops tauschii* | uncharacterized LOC109745284 | 97 | 1.00E-23 | 97.01 | XR_002228514.1 |
| 4R | 3576895 (A, AS) | *Aegilops tauschii* | stem 28 kDa glycoprotein-like | 100 | 4.00E-23 | 95.65 | XM_020321516.1 |
| 4R | 3577197 (A, AS) | *Aegilops tauschii* | uncharacterized LOC109745192 | 56 | 4.00E-04 | 87.18 | XM_020304324.1 |
| 4R | 3577943 (A, AS) | *Aegilops tauschii* | uncharacterized LOC109732755 | 97 | 1.00E-16 | 89.55 | XR_002225459.1 |
| 4R | 3586624 (A, AS) | *Aegilops tauschii* | uncharacterized LOC109782793 | 82 | 3.00E-19 | 98.25 | XM_020341406.1 |
| 4R | 3587751 (A, AS) | *Aegilops tauschii* | uncharacterized LOC109745191 | 69 | 2.00E-07 | 86.27 | XM_020304322.1 |
| 4R | 3590786 (A, AS) | *Aegilops tauschii* | keratin-associated protein 5-5-like | 86 | 6.00E-09 | 77.78 | XM_020293636.1 |
| 4R | 3593377, 3593538 (A, AS) | *Triticum aestivum* | clone: whxk12c03, cultivar Chinese Spring | 97 | 4.00E-11 | 83.58 | AK446222.1 |
| 4R | 3599981 (A, AS) | *Hordeum vulgare* | Rfm1 gene locus, complete sequence | 88 | 3.00E-06 | 80.00 | MF443757.1 |
| 4R | 3600244, 3601082 (A, AS) | *Hordeum vulgare* | mRNA for predicted protein, complete cds, clone: NIASHv1031O22 | 100 | 1.00E-17 | 89.86 | AK356239.1 |
| 4R | 3602801 (A, AS) | *Aegilops tauschii* | DExH-box ATP-dependent RNA helicase DExH12 | 60 | 9.00E-13 | 100.00 | XM_020321763.1 |
| 4R | 3730381 (A, AS) 3730381_7:T>C | **B. distachyon* | uncharacterized LOC100828826 | 68 | 5.00E-10 | 91.49 | XM_010230122.3 |
| 4R | 3731439 (A) | *Aegilops tauschii* | disease resistance protein RPM1-like | 95 | 1.00E-10 | 83.33 | XM_020291268.1 |
| 4R | 3733750 (A, AS) | *Aegilops tauschii* | uncharacterized LOC109784762 | 59 | 4.00E-04 | 85.37 | XM_020343360.1 |
| 4R | 3738456 (A) | *Aegilops tauschii* | UPF0481 protein At3g47200-like | 100 | 3.00E-19 | 91.30 | XM_020298976.1 |
| 4R | 3743168 (A, AS) | *B. distachyon* | uncharacterized LOC112270771 | 92 | 8.00E-07 | 79.69 | XM_024458875.1 |
| 4R | 3746061 (A, AS) | *Aegilops tauschii* | keratin-associated protein 5-5-like | 100 | 2.00E-25 | 97.10 | XM_020293636.1 |
| 4R | 3885888 (A, AS) | *Aegilops tauschii* | transcription termination factor MTERF15, mitochondrial-like | 92 | 2.00E-18 | 92.19 | XM_020340783.1 |
| 4R | 5037479 (A, AS) | *Aegilops tauschii* | keratin-associated protein 5-4-like | 100 | 8.00E-26 | 98.55 | XM_020293637.1 |
| 4R | 16404797 (A, AS) | *Aegilops tauschii* | uncharacterized LOC109767484 | 94 | 1.00E-11 | 84.62 | XM_020326238.1 |
| 4R | 3348274 (A, AS) | *Aegilops tauschii* | keratin-associated protein 5-5-like | 60 | 4.00E-11 | 97.62 | XM_020293636.1 |
| 4R | 5042964 (A, AS) | *B. distachyon* | glycine-rich protein 2 | 97 | 2.00E-07 | 80.00 | XM_003562927.4 |
| 4R | 5201598 (A, AS) | *Oryza sativa* | cultivar Shuhui498 chromosome 4 sequence | 88 | 4.00E-04 | 89.47 | CP018160.1 |
| 4R | 5203263 (A, AS) | *Aegilops tauschii* | uncharacterized LOC109745189 | 97 | 6.00E-21 | 94.03 | XM_020304321.1 |
| 4R | 5208373 (A, AS) | *Triticum aestivum* | dehydration-induced protein (DRPL) | 47 | 7.00E-08 | 100.00 | AY987028.2 |
| 4R | 5217607 (A) | *Hordeum vulgare* | mRNA for predicted protein, complete cds, clone: NIASHv2070F16 | 86 | 1.00E-11 | 86.67 | AK355797.1 |
| 4R | 5223845 (A, AS) | *B. distachyon* | receptor-like protein kinase 5 | 68 | 2.00E-08 | 89.36 | XM_010233998.3 |
| 4R | 5500712 (A, AS) | *Hordeum vulgare* | Rfm1 gene locus, complete sequence | 60 | 3.00E-06 | 90.48 | MF443757.1 |
| 4R | 16404796 (A, AS) | *B. distachyon* | uncharacterized LOC100827785 | 82 | 2.00E-08 | 84.21 | XM_024462438.1 |
| 4R | 3349306 (A, AS) | *Aegilops tauschii* | uncharacterized LOC109745192 | 57 | 6.00E-09 | 95.00 | XM_020304324.1 |
| 4R | 3358170 (A, AS) | *Aegilops tauschii* | uncharacterized LOC109749410 | 76 | 9.00E-19 | 100.00 | XR_002229633.1 |
| 4R | 3358919 (A, AS) | *Aegilops tauschii* | ankyrin repeat-containing protein At5g02620-like | 59 | 2.00E-09 | 95.12 | XM_020338899.1 |
| 4R | 3576311 (A, AS) | *Aegilops tauschii* | uncharacterized LOC109758950 | 95 | 9.00E-19 | 92.42 | XR_002231957.1 |
| 4R | 3577486 (A) | *Aegilops tauschii* | disease resistance protein RPM1-like | 86 | 9.00E-13 | 88.33 | XM_020291268.1 |
| 4R | 3597763 (A, AS) | *Aegilops tauschii* | disease resistance protein RPM1-like | 50 | 1.00E-04 | 91.43 | XM_020291268.1 |
| 4R | 3730066 (A, AS) | *Aegilops tauschii* | uncharacterized LOC109756116 | 100 | 2.00E-20 | 92.75 | XM_020314973.1 |
| 4R | 3738354 (A, AS) | *Aegilops tauschii* | proline-rich protein HaeIII subfamily 1-like | 68 | 4.00E-05 | 85.42 | XM_020296495.1 |
| 4R | 3747675, 3750129 (A, AS) | *Aegilops tauschii* | uncharacterized LOC109784264 | 98 | 3.00E-06 | 77.94 | XM_020342864.1 |
| 4R | 4096992 (A, AS) | *Aegilops tauschii* | keratin-associated protein 5-5-like | 100 | 6.00E-15 | 86.96 | XM_020293636.1 |
| 4R | 5040170 (A, AS) | *Aegilops tauschii* | nitrate reductase [NADH] | 79 | 3.00E-18 | 98.18 | XM_020327107.1 |
| 4R | 7497373 (A, AS) | *Triticum aestivum* | clone: tplb0055n20, cultivar Chinese Spring | 85 | 1.00E-16 | 93.22 | AK448342.1 |
| 4R | 3576571_12:T>C (A, AS) | *Aegilops tauschii* | uncharacterized LOC109784158 | 79 | 1.00E-04 | 80.00 | XM_020342751.1 |
| 4R | 3584270_26:A>C (A, AS) | *Aegilops tauschii* | putative disease resistance RPP13-like protein 3 | 86 | 9.00E-13 | 88.33 | XM_020304319.1 |
| 5R | **5036750 (S)** | *-* | n.a. |  |  |  |  |
| 5R | 3356644_14:C>G (A) | *Triticum aestivum* | aquaporin (PIP2-11) gene | 94 | 2.00E-15 | 89.23 | EU177564.1 |
| 5R | 3585163_8:G>A (A) | *Aegilops tauschii* | probable aldo-keto reductase 2 | 79 | 4.00E-17 | 96.36 | XM_020295550.1 |
| 5R | 7467673 (A) | *B. distachyon* | uncharacterized LOC104585133 | 73 | 3.00E-06 | 83.33 | XM_014902552.2 |
| 5R | 3742143 (A) | *Hordeum vulgare* | mRNA for predicted protein, complete cds, clone: NIASHv1004P08 | 78 | 1.00E-10 | 89.09 | AK354091.1 |
| 5R | 5212120_43:A>G (A) | *Oryza sativa* | probable aldo-keto reductase 2 | 100 | 1.00E-24 | 97.10 | XM_015780469.2 |
| 5R | 3887988 (A) | *Aegilops tauschii* | uncharacterized LOC109736345 | 100 | 8.00E-20 | 90.41 | XM_020295568.1 |
| 5R | **3601104 (S)** | *-* | n.a. |  |  |  |  |
| 5R | 3587839 (A) | *Aegilops tauschii* | thaumatin-like protein 1b | 81 | 1.00E-17 | 96.43 | XM_020299767.1 |
| 5R | **4498362 (S)** | *-* | n.a. |  |  |  |  |
| 5R | 3342917 (R) | *Aegilops tauschii* | putative disease resistance RPP13-like protein 2 | 100 | 4.00E-23 | 95.65 | XM_020313763.1 |
| 5R | 3357941 (R) | *Zea mays* | putative disease resistance protein At1g50180 | 52 | 8.00E-07 | 94.44 | XM_008681141.2 |
| 5R | 3342019 (R) | *Aegilops tauschii* | putative disease resistance RPP13-like protein 3 | 100 | 1.00E-24 | 97.10 | XM_020313758.1 |
| 5R | 5504657 (R) | *Hordeum vulgare* | clone: tplb0045f07, cultivar Chinese Spring | 72 | 9.00E-13 | 94.00 | AK451238.1 |
| 5R | 3341963 (R) | *Triticum aestivum* | clone: WT009_D05, cultivar: Chinese Spring | 44 | 4.00E-04 | 93.55 | AK334273.1 |
| 5R | 7097204 (A) | *Aegilops tauschii* | proline-rich protein 36-like | 98 | 6.00E-09 | 80.88 | XM_020307307.1 |
| 5R | 3344087 (A) | *Aegilops tauschii* | uncharacterized LOC109752758 | 92 | 1.00E-17 | 92.19 | XM_020311651.1 |
| 5R | 3344746 (A) | *Hordeum vulgare* | mRNA for predicted protein, complete cds, clone: NIASHv3083D19 | 95 | 2.00E-07 | 80.60 | AK375008.1 |
| 5R | 4494460 (A) | *Triticum aestivum* | clone: tplb0045f07, cultivar Chinese Spring | 97 | 2.00E-07 | 79.10 | AK451238.1 |
| 5R | 3350404 (A) | *Triticum aestivum* | clone: tplb0045f07, cultivar Chinese Spring | 100 | 3.00E-12 | 84.06 | AK451238.1 |
| 5R | **3581763 (S)** | *Aegilops tauschii* | thaumatin-like protein 1b | 84 | 4.00E-17 | 94.83 | XM_020332140.1 |
| 5R | 4092866 (R) | *Aegilops tauschii* | serine/threonine protein phosphatase 2A 57 kDa regulatory subunit B' alpha isoform-like | 100 | 8.00E-26 | 98.55 | XM_020332099.1 |
| 5R | 6210283_19:G>C (A) | *Aegilops tauschii* | NEP1-interacting protein 2-like | 43 | 1.00E-04 | 96.67 | XM_020332142.1 |
| 5R | 3748078 (A) | *Aegilops tauschii* | NEP1-interacting protein 2-like | 69 | 1.00E-10 | 91.67 | XM_020332142.1 |
| Markers associated (**AS**)^#^ with the pollen fertility restoration locus, not localized on the map | | | | | | | |
| n.a. | 3582921 | *Aegilops tauschii* | PREDICTED: uncharacterized LOC109767484 | 53 | 2.00E-07 | 94 | XM_020326238.1 |
| n.a. | 3600245 | *Hordeum vulgare* | complete cds, clone: NIASHv1031O22 | 100 | 1.00E-17 | 90 | AK356239.1 |
| 4R | 3362765_16:T>A | *Hordeum vulgare* | Rfm1 gene locus, partial sequence | 73 | 1.00E-17 | 100 | MF443756.1 |
| n.a. | 3894081 | *Aegilops tauschii* | uncharacterized LOC109766270 | 100 | 2.00E-27 | 100.00 | XR_002233579.1 |
| n.a. | 16404806 | *Triticum aestivum* | gamma gliadin-B1, gamma gliadin-B2, delta gliadin-B1 | 69 | 4.00E-04 | 83.33 | MG560141.1 |
| 4R | 3588282_8:A>G | *Aegilops tauschii* | uncharacterized LOC109745191 | 73 | 5.00E-16 | 98.04 | XM_020304322.1 |
| 4R | 4094880_16:A>T | *Triticum aestivum* | omega gliadin-D1, omega gliadin-D2, omega gliadin-D3 | 78 | 8.00E-07 | 83.33 | MG560142.1 |
| 4R | 7093267_30:C>T | *Triticum aestivum* | clone: WT010_P23, cultivar: Chinese Spring | 50 | 3.00E-06 | 94.29 | AK334770.1 |
| n.a. | 3582000 | *Aegilops tauschii* | uncharacterized LOC109776519 | 100 | 2.00E-20 | 92.75 | XM_020335162.1 |
| n.a. | 3591308 | *Triticum aestivum* | clone: tplb0060i15, cultivar Chinese Spring | 100 | 4.00E-11 | 82.61 | AK452918.1 |
| n.a. | 5138870 | *Hordeum vulgare* | mRNA for predicted protein, complete cds, clone: NIASHv1026A19 | 97 | 1.00E-16 | 89.55 | AK355797.1 |
| n.a. | 5221291 | *B. distachyon* | uncharacterized LOC112270771 | 92 | 2.00E-08 | 81.25 | XM_024458875.1 |
| n.a. | 3744939 | *B. distachyon* | uncharacterized LOC104583403 | 100 | 1.00E-16 | 88.41 | XM_024462439.1 |
| n.a. | 3589890 | *Aegilops tauschii* | disease resistance RPP13-like protein 3 | 79 | 4.00E-17 | 96.36 | XM_020304320.1 |
| n.a. | 3591564 | *Aegilops tauschii* | putative cyclin-dependent kinase F-2 | 60 | 2.00E-08 | 92.86 | XM_020342573.1 |
| n.a. | 5208386 | *Aegilops tauschii* | receptor-like protein kinase 5 | 100 | 4.00E-11 | 82.61 | XM_010233998.3 |
| 4R | 16520239 | *Aegilops tauschii* | cytochrome P450 709B2-like | 100 | 8.00E-26 | 98.55 | XM_020296917.1 |

* - *Brachypodium distachyon*

# - only markers with the highest association coefficient values (R^2^ ≥0.44) are listed

ª - not assigned
